# Health Care and Precision Medicine Research: Analysis of a Scalable Data Science Platform

**DOI:** 10.2196/13043

**Published:** 2019-04-09

**Authors:** Jacob McPadden, Thomas JS Durant, Dustin R Bunch, Andreas Coppi, Nathaniel Price, Kris Rodgerson, Charles J Torre Jr, William Byron, Allen L Hsiao, Harlan M Krumholz, Wade L Schulz

**Affiliations:** 1 Department of Pediatrics Yale University School of Medicine New Haven, CT United States; 2 Department of Laboratory Medicine Yale University School of Medicine New Haven, CT United States; 3 Center for Outcomes Research and Evaluation Yale-New Haven Hospital New Haven, CT United States; 4 Yale New Haven Health Information Technology Services New Haven, CT United States; 5 Section of Cardiovascular Medicine Department of Internal Medicine Yale School of Medicine New Haven, CT United States; 6 Department of Health Policy and Management Yale School of Public Health New Haven, CT United States

**Keywords:** data science, monitoring, physiologic, computational health care, medical informatics computing, big data

## Abstract

**Background:**

Health care data are increasing in volume and complexity. Storing and analyzing these data to implement precision medicine initiatives and data-driven research has exceeded the capabilities of traditional computer systems. Modern big data platforms must be adapted to the specific demands of health care and designed for scalability and growth.

**Objective:**

The objectives of our study were to (1) demonstrate the implementation of a data science platform built on open source technology within a large, academic health care system and (2) describe 2 computational health care applications built on such a platform.

**Methods:**

We deployed a data science platform based on several open source technologies to support real-time, big data workloads. We developed data-acquisition workflows for Apache Storm and NiFi in Java and Python to capture patient monitoring and laboratory data for downstream analytics.

**Results:**

Emerging data management approaches, along with open source technologies such as Hadoop, can be used to create integrated data lakes to store large, real-time datasets. This infrastructure also provides a robust analytics platform where health care and biomedical research data can be analyzed in near real time for precision medicine and computational health care use cases.

**Conclusions:**

The implementation and use of integrated data science platforms offer organizations the opportunity to combine traditional datasets, including data from the electronic health record, with emerging big data sources, such as continuous patient monitoring and real-time laboratory results. These platforms can enable cost-effective and scalable analytics for the information that will be key to the delivery of precision medicine initiatives. Organizations that can take advantage of the technical advances found in data science platforms will have the opportunity to provide comprehensive access to health care data for computational health care and precision medicine research.

## Introduction

### Background

Health care has seen massive data growth over the last several years, with some reports estimating that health care data generation increases by 48% annually [1]. In addition, it has been estimated that the intelligent use of big data within the health care sector in the United States could save over US $300 billion [2]. One particular area of medicine that relies heavily on these big data sources is precision medicine, where massive amounts of information are needed to provide precision diagnostics or therapeutics [3-5]. However, efforts to store, manage, and analyze these growing datasets have stretched the limits of traditional health care information technology systems [6].

Many definitions of big data exist, with one of the simplest being “any dataset that is too large or complex for traditional hardware or data management tools” [7]. In addition to the significant increases in volume, health care data are highly complex due to the presence of many data standards and an estimated 80% of information being unstructured [8]. These data can be problematic for traditional enterprise solutions, which rely heavily on defined data models prior to indexing, making it difficult to accommodate new data feeds or evolving data structures [9]. To support the informatics needs for the next generation of computational health research, novel approaches to data storage and analysis are necessary.

Fortunately, several applications have emerged that begin to address the key challenges in big data processing, such as distributed data storage and scalable processing capacity [10]. One example is the Hadoop platform, a set of open source tools designed specifically for these tasks [11]. The goal of these platforms is to create a central repository, called a data lake, which can store raw data in its native format for later search, retrieval, and analysis. However, researchers and clinicians in the health care sector looking to leverage modern big data architectures are faced with particular challenges in implementation and little guidance or evidence on the use of these platforms in parallel with production environments.

With the push for population-wide research initiatives in the United States such as the Cancer Moonshot [12] and Precision Medicine Initiative (now called All of Us) [3] that will rely on large, complex, interrelated data, institutions need to develop systems that can adequately scale to handle the data influx and provide sufficient capacity for analytic needs. Nevertheless, any new approaches must be attentive to the privacy and reliability requirements associated with health care data. Accordingly, we present 2 use cases that highlight the architecture and implementation of a health care data science platform that enables integrated, scalable, secure, and private health care analytics. These strategies highlight current best practices for data management, system integration, and distributed computing, while maintaining a high level of security and reliability.

### Objective

We describe how an integrated data lake and analytics platform can be used to provide near real-time access to health care and biomedical research data and the ability to conduct computational health care research. We describe the implementation of such a platform, which we have named the Yale New Haven Health Baikal Data Science Platform. We highlight the data workflows and use of specialized data storage formats for 2 common health care use cases: continuous patient monitoring and real-time laboratory analytics. We chose these because they use large, real-time datasets that are difficult to store in traditional health care data warehouses.

## Methods

### Hardware and Operating Systems

We deployed the Hadoop platform (Apache Software Foundation) on a 30-node cluster running CentOS7 (Red Hat). No virtualization layer was used so as to minimize performance overhead. This cluster has a total of 552 processing cores, 9 TB of memory, and 714 TB of storage distributed among the nodes, with a scalable framework that can be used to add additional capacity. We deployed 5 additional nodes running CentOS7 with a distributed total of 60 cores, 320 GB of memory, and 5 TB of storage to run Elasticsearch (Elasticsearch BV). In addition to the core data storage and analysis nodes, we created 4 virtual application servers: 2 running CentOS7 and 2 running Windows Server 2012 R2 (Microsoft Corporation). We also deployed a virtual machine running CentOS7 as the Ambari management node (Apache Software Foundation) for the Hadoop cluster.

### Software and System Configuration

We installed Hortonworks Data Platform version 2.6.0, a commercially supported Hadoop distribution (Hortonworks Inc), with 3 master nodes, 3 edge nodes, and 24 data nodes. We deployed Ambari with Ansible (Red Hat) playbooks and individual Hadoop applications deployed through the Ambari interface. We installed key software packages from Hortonworks Data Platform version 2.6.0, including the Hadoop Distributed File System (HDFS), Zookeeper, Yet Another Resource Negotiator (more commonly known as YARN), Kafka, Storm, and Spark, on these nodes, in high-availability mode when possible. We deployed Docker (Docker Inc) within a Swarm configuration on the 3 edge nodes. We deployed Hortonworks Data Flow (HDF) version 3.0 (Hortonworks Inc), based on the open source NiFi software (version 1.2.0.3), within a Docker container on 1 edge node.

We deployed Elasticsearch version 6.2.2 within Docker containers to 5 individual nodes. We configured 1 node as a dedicated master node, 2 nodes as master-eligible data nodes, and 2 data nodes. We deployed Kibana version 6.2.2 (Elasticsearch BV) in a Docker container to 1 Linux application server. Other software components relevant to the use cases discussed here include version 3.6 of the RabbitMQ software (Pivotal Software, Inc), the Capsule Neuron software (Qualcomm Life, Inc), and the Cloverleaf (Infor) interface and integration engine.

Physiologic monitoring data were validated by a team of nurses, respiratory therapists, engineers, and physicians simultaneously reviewing specific bedside monitor results with the real-time data feed in the data science platform. Random provider-validated data points within the electronic health record were also compared with values captured by the data science platform. Laboratory data feeds were similarly validated by physician review of randomly selected laboratory orders and results, as well as by comparison of result counts in the reference-standard clinical data warehouse and data science platform.

### Compression Efficiency Assessment

We developed a Spark application in the Scala programming language to compare the storage and analytic efficiency of 3 file formats: standard comma-separated values, Avro, and data compressed with the Snappy codec (Google LLC). We extracted data for a 1-month monitoring period for performance testing. We loaded data elements and then wrote them to HDFS in each file format. We obtained the execution time for the read and write efficiency from the Spark shell interface. We repeated this process on 3 separate edge nodes and calculated the mean execution time for each measurement.

## Results

### Platform Architecture and Deployment

#### Core Components

**Figure 1:**
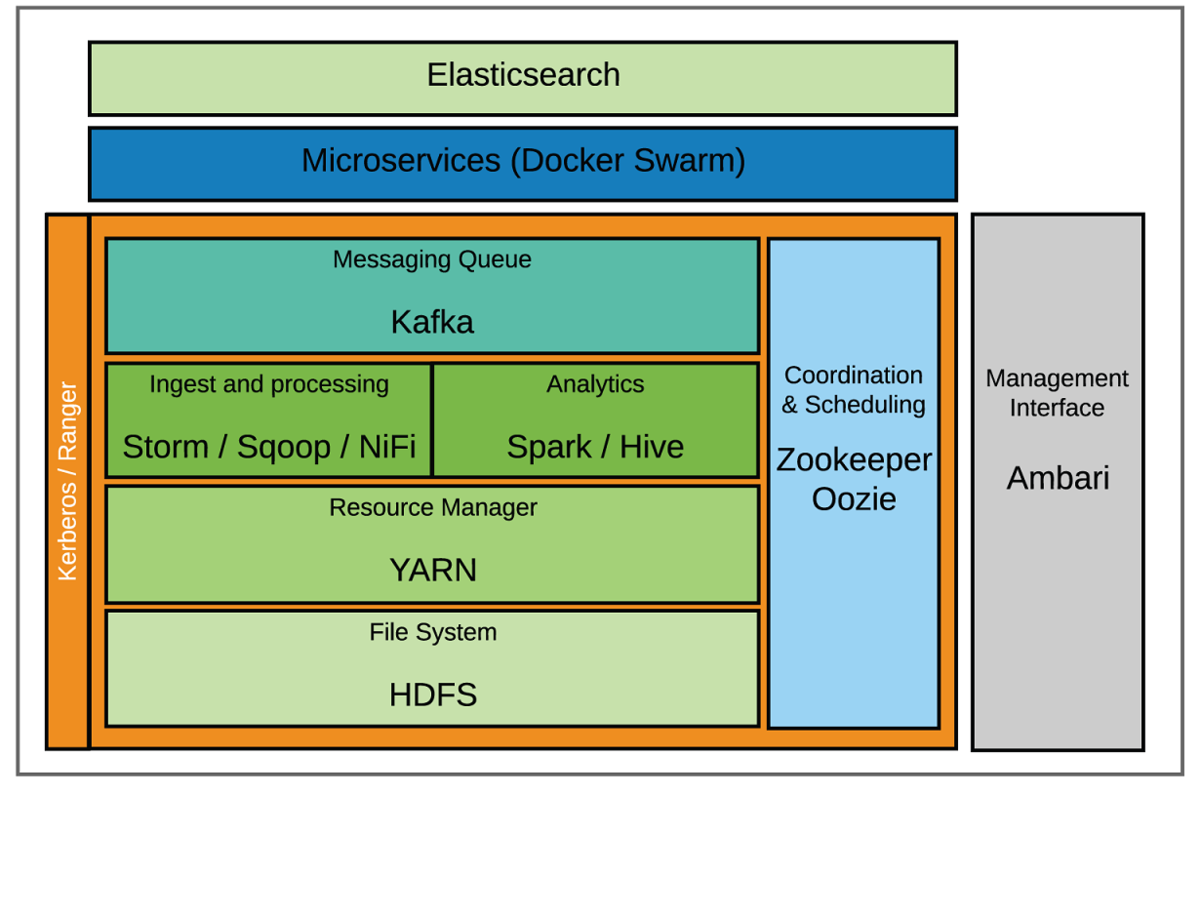
Baikal platform architecture. Cluster services are monitored, deployed, and provisioned by Ambari management console. Workflow management and configuration synchronization are handled by Zookeeper and Oozie. Data storage frameworks include Hadoop Distributed File System (HDFS) and a nonrelational database: Elasticsearch. Kafka messaging queues are used for incoming data with subsequent ingest and processing handled by Storm, Sqoop, and NiFi. Analytics can be performed by Spark and Hive. Kerberos and Ranger are used to secure cluster applications. Lastly, Docker Swarm is used to deploy custom applications that can be run within the data science platform. YARN: Yet Another Resource Negotiator.

The core software applications within the Baikal platform include features that allow for distributed data storage, message queuing, streaming data processing, distributed computation, and workflow management (Figure 1). Two key systems form the basis of the data storage platform: HDFS and Elasticsearch, a NoSQL database platform. Two message queue applications are also used within the data science platform. Kafka is used within the Hadoop environment and RabbitMQ is used on nodes outside of the Hadoop cluster. Streaming data are processed with Storm, a distributed real-time computation system, or HDF, which provides similar features but with a developer-friendly user interface. Distributed batch computation is done with the Spark framework and custom applications. Workflow management and configuration synchronization are done with the Oozie and Zookeeper applications, respectively. Finally, Docker is used for the deployment of custom applications that can be run within the data science platform.

**Table 1:** Average storage requirements for adult and pediatric patient monitoring and ventilator monitoring. Signal frequency and storage size are the metrics for a complete 24-hour per-bed monitoring period averaged from 3 independent samples.

Source	Signal frequency, mean (SD)	Storage size (MB), mean (SD)	Estimated annual storage (GB)
Adult monitor	291,252 (84,568)	17.1 (5.0)	6.2
Pediatric monitor	223,387 (29,543)	12.7 (1.8)	4.6
Adult ventilator	3,504,162 (236,672)	231.5 (30.6)	84.5

#### Security

Many big data platforms, including Hadoop, have limited security features enabled by default [13,14]. For example, no authentication is required to access Web service interfaces by default in either Hadoop or Elasticsearch [14,15]. This lack of default security has led to several data breaches over the last several years [16]. Fortunately, these platforms do allow for configuration of a robust security system with the use of Kerberos, Ranger, and Shield [13,17]. Within the Baikal platform, we deployed a dedicated Kerberos realm for authentication into the cluster. We deployed Ranger to allow for permission-based authorization to resources in the cluster at both the application and data layers.

### Electron: A Framework for Physiologic Signal Monitoring and Analysis

Continuous monitoring of patient vital signs has been standard practice in intensive care units and emergency departments. However, these data are rarely kept longer than a few days due to the storage and technical requirements for such large datasets with limited impact for clinical use; however, they may have significant value for future investigation. To support investigators who require access to these physiologic signals, we used the Baikal platform to create Electron, a framework to store and analyze longitudinal physiologic monitoring data. The code for this platform is available within a GitHub repository [18].

#### Data Characteristics

Many bedside patient monitors and ventilators are able to transmit their settings and recordings to a central application at specific intervals. Within our institution, these signals are sent at 1- to 5-second intervals, depending on the device, data element, and value. These data elements include active data channels, device and patient metadata, and more intermittent data elements, such as noninvasive blood pressure and alarms. In total, data can be transmitted for up to 892 active and metadata channels for bedside monitors and 45 channels for ventilators. Individual message size varies based on the number of metadata elements, the device being used for monitoring, and frequency of intermittent events. To determine the data storage requirements for such a platform, we collected data for 3 randomly selected adult and 3 pediatric patients in the intensive care unit for a 24-hour time frame. A single adult patient in the intensive care unit generated approximately 17.1 MB of data per 24-hour time frame (Table 1).

Similarly, a 24-hour monitoring period for pediatric patients averaged approximately 12.7 MB in the same time frame. We similarly assessed the data volume generated by ventilators in our health system, which produced approximately 231.5 MB of data per ventilator per day. When we assessed monitoring data from a 1-month period, we collected over 6 TB of raw data from 11 units and a total of 225 beds, often reaching rates of over 400 messages/s. These units were diverse and included intensive care, surgical, emergency department, and short-stay beds.

#### Electron Framework Architecture

**Figure 2:**
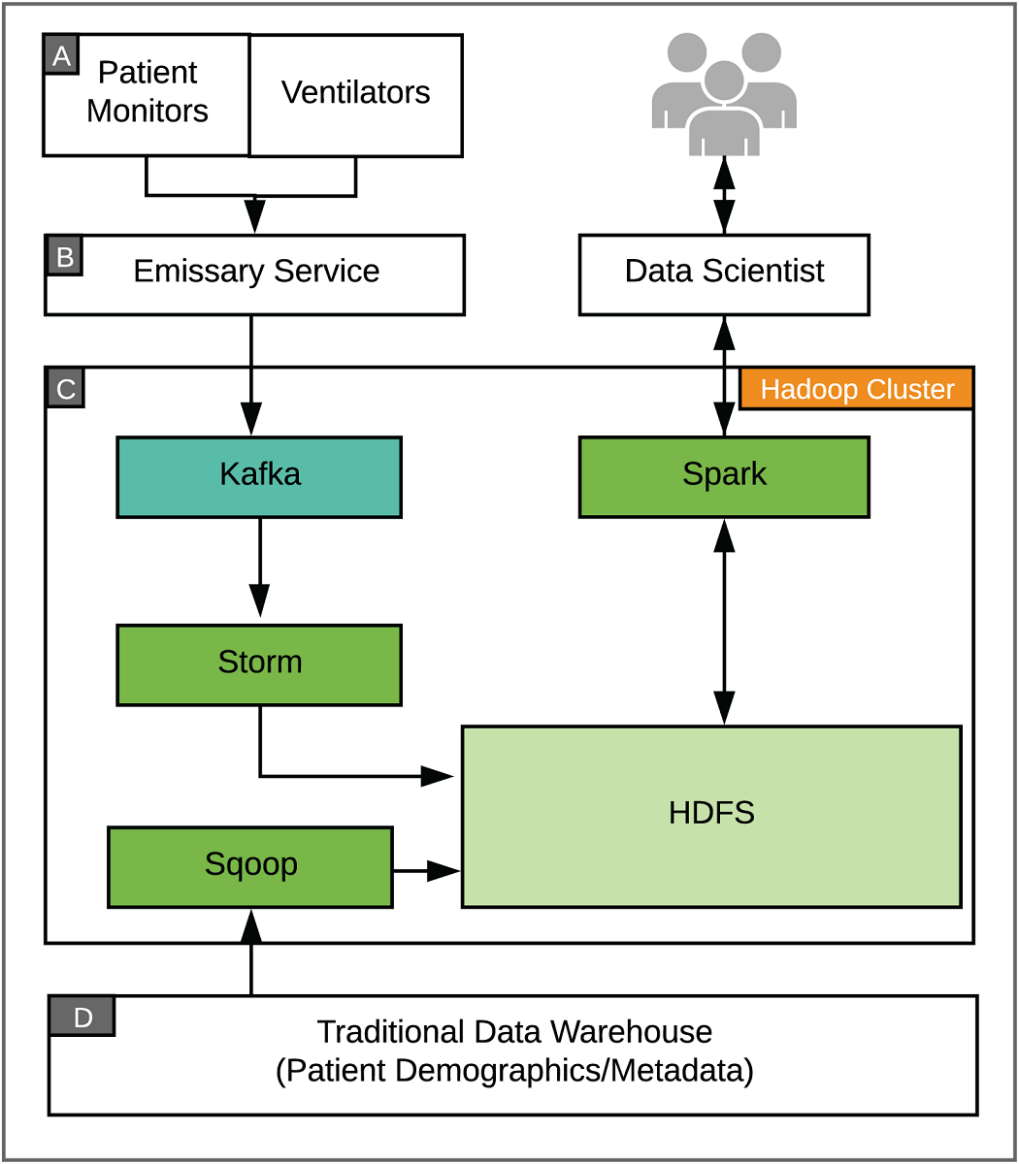
System architecture for continuous patient monitoring. Multiple, increasing sources of clinical data (A) acquire and transmit the data to aggregation servers, which then forward Health-Level 7 (HL7) messages to an emissary service (B), where data are normalized and securely forwarded in standardized JSON format to the Baikal system (C) for denormalization, processing, and storage in the Hadoop Distributed File System (HDFS). Traditional historic databases (D) are individually prepared for ingestion in the Baikal system and storage in HDFS. The resulting data lake allows for integrated, distributed analytics by end users.

The platform to acquire, store, and analyze the continuous monitoring data consists of 4 key features: data ingestion, data processing and denormalization, compressed storage, and distributed analytics (Figure 2). Our physiologic monitoring infrastructure consists of attached patient monitoring devices that send signals to vendor-supported integration servers (Figure 2, box A). Data are then transmitted as Health-Level 7 (HL7) messages streamed via a Transmission Control Protocol/Internet Protocol connection to an emissary service that we deployed to accept the incoming message stream and perform the initial conversion of HL7 messages into a custom JSON format (Figure 2, box B). Date and time information is converted to Coordinated Universal Time (UTC), while all other data are left in their original format. Once processed, messages are forwarded to a secured Kafka message queue, which allows the platform to buffer messages during downstream processor downtime or when under heavy load. The JSON document contains key elements for downstream processing, as well as a copy of the original HL7 message to allow for future reprocessing, if needed (Textbox 1).

The decision to store the original data is often considered a best practice but has the disadvantage of increasing the storage requirements for the dataset. While we opted to store the raw data on the platform, as the HL7 message contains additional information that may be needed for future studies, the decision to maintain this information can be made for each use case dependent on the data storage costs and estimated future utility of the information.

**JSON data model for physiologic data.**

{

“msh_ts”:“long”,

“alarm_ts”:“long”,

“source”:"string",

“unit”:“string”,

“text”:“string”,

“channel”:“string”,

“hl7”:“string”

}

**Figure 3:**
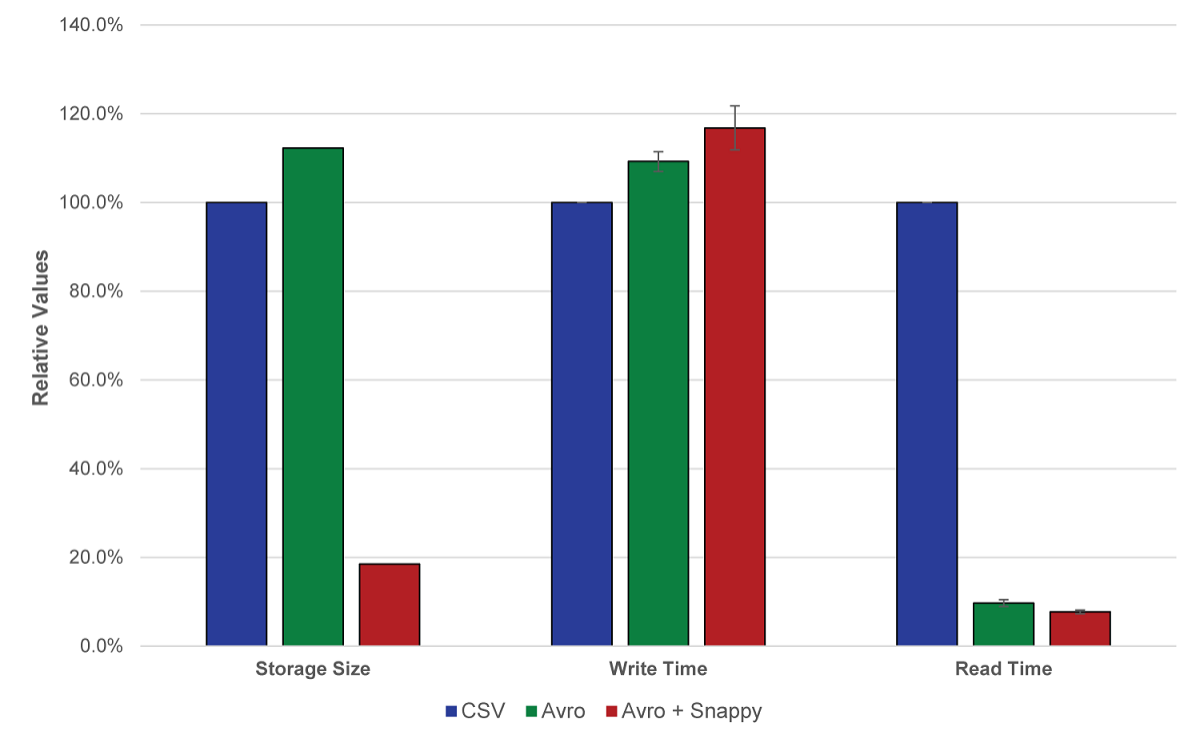
Comparison of storage and read/write efficiency. Avro increases storage space and write time modestly while significantly reducing read time. The addition of Snappy compression increases write time minimally, while significantly decreasing storage space and maintaining minimal read time. The resulting combination optimizes for single archival write with multiple read usage. CSV: comma-separated values. Error bars represent standard error.

#### Storage Formats and Data Processing

While storage costs continue to decline, the cost of long-term data storage for large datasets remains burdensome. Specialized data formats and compression can improve the density of data storage but often come at the cost of increased overhead for read and write throughput. Fortunately, the frequency of access to historic data typically decreases over time, which means that slower data access methods would have less impact on overall analytic capacity. Other work has compared the storage and access efficiency for many big data technologies [19]. For this use case, we predicted that the Avro data format with Snappy compression would have an appropriate balance of storage and access efficiency.

Avro is a semi-structured data serialization format designed for big data storage. In addition to the semi-structured nature of the Avro format, the files are also splittable, which means that the Hadoop platform can separate the file into individual sections, which increases the processing efficiency during data analysis [20]. To assess the impact of the Avro format and Snappy compression, we assessed the storage and access efficiency of monitoring data from several different variables over a 30-day period in comma-separated raw text, Avro, and Snappy-compressed Avro formats. Data were filtered and the length of time needed to write and read data from 3 independent nodes in the cluster was recorded. Compared with raw text, Avro-formatted files required approximately 12% more storage space on disk but showed significantly faster data retrieval time (Figure 3). The use of Snappy compression showed significant savings in storage requirements, with an average reduction in file size of 80.5% compared with raw text files. Also of note was the large reduction in time needed to access data stored in Snappy-compressed Avro files.

In addition to the large volume, the high velocity of these data required a high-throughput data processing pipeline to convert and store the data efficiently. To achieve this, we developed a custom application built on the Storm platform that allowed for distributed, high-throughput processing. Within the Storm topology, monitor signals were denormalized, converted to the Avro format, compressed with the Snappy codec, and stored in HDFS to allow for future analysis. A separate copy of the data containing the original HL7 message was also stored through a separate Storm bolt in case reprocessing of the raw data became necessary.

#### Analytics

Much like the particular challenges for the acquisition and storage of big data, specialized needs for the analysis of these datasets also exist. While the raw data are of use for many research and clinical projects, derived variables and predictive analytics are often of interest. For example, computationally derived features, such as R-R intervals [21], indices of multiple vital signs [22], and temporal relations between vital signs have all shown promise as predictive variables [23]. However, generating these features is often computationally intensive when performed at scale on entire patient populations.

Traditional analytic methods and tools are often unable to scale to meet the needs of these analyses. Even in cases where parallelized computation can be used, the resources necessary to develop and validate these custom applications is often prohibitive. To make parallelized computation more accessible, solutions such as MapReduce [11] and Dryad [24] have been created, which provide frameworks that manage the complexity of parallelization. However, these solutions still require significant technical expertise to develop applications that can be deployed to production environments. Within the Baikal platform, we enabled Spark as the primary data analysis tool for batch analysis. Spark is a general data processing framework that can be used to write applications in several common languages, including Java, Scala, Python, and R. A key advantage of this framework is the ability to maintain data for MapReduce operations in memory, rather than needing to read and write each intermediate step to disk. This has been shown to improve the speed of big data processing significantly [25,26]. We developed several Spark applications that can be used by data analysts to generate features from the physiologic data, such as alarm events, and extract subsets of information for downstream processing, which are available within the GitHub repository.

While the physiologic data are captured during routine clinical care, a major goal of this dataset is for use in biomedical research. Because of the sensitive nature and regulatory oversight of human subjects research, appropriate approvals, such as institutional review board approval and patient consent, are needed prior to the analysis of data within the system. In addition, our clinical data warehouse (Figure 2, box D) includes a field that indicates whether patients have explicitly opted out of research and is used to filter their data from analysis that is classified as human subjects research.

#### Nucleus: A Platform for Real-Time Laboratory Business Intelligence and Data Visualization

In addition to novel data sources such as continuous patient monitoring, data science platforms can also offer new approaches for the analysis of more traditional health care datasets. Examples include real-time data analysis, predictive analytics, and interactive visualizations. In the era of cost reduction and an increasing demand for clinical laboratory services, laboratorians are facing expectations to optimize laboratory efficiency for the sake of clinical workflows and improve test utilization without compromising quality and safety. Therefore, the clinical laboratory has a particular need for real-time business intelligence to improve testing efficiency and patient safety [27]. To achieve this, we created a data science platform with business intelligence dashboards to monitor testing within our institution’s clinical laboratory [28].

##### Data Characteristics

Laboratory orders and results are often routed through multiple systems as they transit between the electronic health record and laboratory instrumentation. This typically includes message integration services and middleware platforms that manage the flow of data between systems created by a number of different vendors. Within our health care system, approximately 40 million individual results are generated annually from 6 hospitals, 26 satellite locations, and approximately 220 laboratory instruments. A principal challenge for these data is to provide real-time access and visualizations to end users who need actionable insights from these disparate systems. Because of these unique needs, many downstream architectural decisions varied from the continuous monitoring application described.

#### Nucleus Platform Architecture

**Figure 4:**
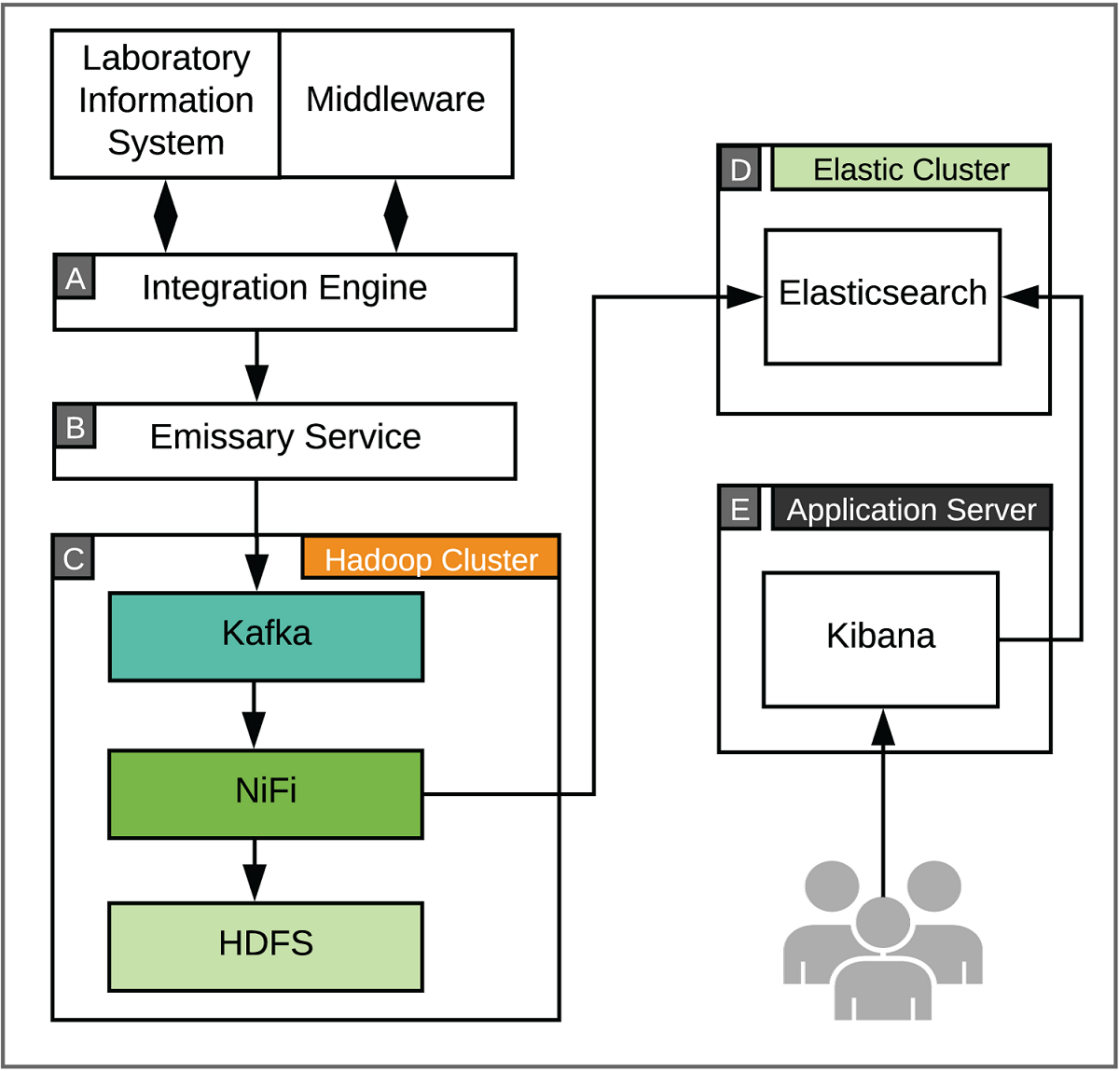
System architecture for laboratory data monitoring. Health-Level 7 (HL7) observations and results messages generated by laboratory information system and laboratory middleware systems are received by the clinical integration engine Cloverleaf (A). HL7 messages are received and validated by a custom emissary service (B) and mapped to JSON documents, which are submitted to a Kafka message queue for downstream processing (C). Custom Python (version 2.7) scripts are executed in NiFi to denormalize messages and calculate quality improvement metrics. Raw HL7 messages are stored in a Hadoop Distributed File System (HDFS). Processed messages and quality improvement metrics are routed to Elasticsearch (D) for real-time analysis and Kibana (E) for visualization.

The initial acquisition of data for this stream is similar to that of the continuous monitoring workflow (Figure 4). Briefly, we deployed an emissary service to receive an HL7 stream of clinical observations and results messages from the Cloverleaf integration engine. Each HL7 message was validated and mapped to a JSON document by the emissary service, then forwarded to a secured Kafka message queue. The custom JSON messages contain key parameters that can be used to index and parse results during batch analysis (Textbox 2).

Because of the slower message velocity, this data stream was easily processed with the NiFi/HDF software, which is designed for real-time data processing. We created custom Python scripts to process and denormalize the incoming data stream. Each order and result message was then written to HDFS for permanent storage and batch analysis and also routed to Elasticsearch for real-time analysis and visualization. Additional features that provide key indicators of laboratory efficiency were generated in real time from the HL7 messages with custom Python scripts that are executed within the NiFi workflow. These quality indicators are stored within Elasticsearch and can be used to visualize turnaround time for laboratory results, outstanding orders, and order volumes by patient or laboratory location.

**JSON data model for laboratory data.**

{

“msh_ts”:"long",

“pt_mrn”:“string”,

“order_id”:“string”,

“lab_type_code”:“string”,

“order_ts”:"long",

“hl7”:“string”

}

## Discussion

### Principal Findings

Health care information is inherently complex and often has an evolving data structure; much of the data is not stored in the electronic health record. Because of this, novel approaches to data management are needed to integrate the many sources of health care data. In addition, novel approaches to data analysis such as machine learning require significant computational resources for timely analysis. As the use of big data in health care continues to increase, the implementation of robust technical solutions to manage and analyze the data will be important to the success of biomedical big data research [3].

In this paper, we have presented the successful implementation of a data science platform along with 2 domain-specific applications deployed within this platform. These applications focused on the storage of high volume, real-time datasets that challenge traditional data warehousing strategies due to their volume and velocity. We have also presented the hardware and architectural approaches used to manage these data. While individual components of the platform used here are described in the nonmedical literature, this platform combines available technologies to meet the known challenges of big data with needs specific to health care, including the security and privacy needs of personal health information.

Often, a single technical solution is unable to address all concerns or needs for a robust data science environment. For example, Hadoop has traditionally been used as a platform for big data storage and batch analysis but had fewer tools available for streaming data and real-time analytics. Because of this, we integrated components designed specifically for the management and visualization of real-time data. This integration allows us to provide efficient batch analytics, as well as real-time visualizations, which would be challenging if only a single tool or platform were used. It should be noted, however, that the applications described here are rapidly evolving and significant strides have been made to expand the features of each component, which may add redundancy between applications in the future.

Data science platforms such as Hadoop offer many individual components to address key requirements for data replication, availability, and security at each stage of the data life cycle, from acquisition to analysis. Fully implementing each of these utilities can make data science pipelines complex, but the use of service-oriented architectures affords the ability to update individual applications, scale services, and reuse individual components in multiple workflows. Because of these rapid developments and the diversity of data, careful testing should be done during the implementation of data science workflows to determine the storage and compute the capacity required for long-term management of the data being obtained. Similarly, careful attention should be paid to the implementation of built-in security features to ensure that data are not accidentally made available to unauthorized users [13].

### Limitations

While data science platforms offer significant potential for the rapid analysis of big data, several limitations exist. In particular, the complexity of these platforms often requires substantial technical expertise to use them to their full potential. Multiple software applications are often needed to implement an entire workflow, particularly within the Hadoop environment. While each Hadoop component often provides significant advantages from developing new applications, personnel with expertise are needed to implement these technologies effectively. While many attempts have been made to make the environment fluent with other tools, such as Python, SAS, and R, seamless integration with these tools remains difficult, particularly in secured environments.

Massive resources have been dedicated to big data and data science in other industries; however, the return on investment has not always been realized. Therefore, the ultimate success of these platforms for computational health research will depend on the ability of the biomedical research community to apply big data to translational and clinical research. Successful application of these technologies with applications that can provide actionable insights from real-world data has the potential to deliver precision medicine at the point of care, but additional studies will be needed to fully assess the impact of these systems on health care delivery and clinical outcomes.

### Conclusion

The paucity of literature describing implementation experiences leaves those interested in developing big data environments largely unguided, particularly within the health care sector, which has unique data and regulatory requirements. Careful attention to the architecture used to create these data science environments will provide an important foundation for future studies that create value from big data sources. As the volume and velocity of health care data continue to increase, additional analyses on the management of these data will be required to ensure that the highest-quality data are made available to efficient analytic systems.

## Abbreviations

HDF: Hortonworks Data Flow
HDFS: Hadoop Distributed File System
HL7: Health-Level 7
YARN: Yet Another Resource Negotiator
